# Running Scared? A Critical Analysis of LGBTQ+ Inclusion Policy in Schools

**DOI:** 10.3389/fsoc.2021.613283

**Published:** 2021-06-09

**Authors:** Jonathan Glazzard, Samuel Stones

**Affiliations:** Carnegie School of Education, Leeds Beckett University, Leeds, United Kingdom

**Keywords:** sexual orientation, LGBTQ+, schools, education, relationships and sex education

## Abstract

This article provides an overview of the UK government policy in relation to relationships and sex education in schools. It focuses on the latest statutory guidance which requires primary and secondary schools in England to teach pupils about different types of relationships, including same-sex relationships. We outline the current policy frameworks and present a rationale for why Lesbian, Gay, Bisexual, Trans and Queer (LGBTQ+) identities and relationships should be present in the curriculum. We critically interrogate the government response and we present a framework to support the implementation of a whole school approach to LGBTQ+ inclusion. We draw on Meyer's model of minority stress to explore risks to children and young people if they are not provided with an LGBTQ+ curriculum.

## Introduction

In 2019 the UK government released statutory guidance for relationships and sex education in schools (Department for Education, 2019) following a period of consultation. The guidance was a radical update of previous guidance which was issued in 2000 to more accurately reflect societal issues in the twenty-first century. The guidance included a requirement for primary and secondary schools to teach children about LGBTQ+ identities and different kinds of relationships, including same-sex relationships. Although societal attitudes in relation to same-sex relationships have improved in recent years, and even though some countries have taken steps to legalize same-sex relationships, the inclusion of this content in the school curriculum was considered by some to be controversial. For example, in 2019 parental opposition in Birmingham and other cities to LGBTQ+ curricula in primary schools dominated the media headlines in England. The apparent tensions between religious beliefs, sexual orientation and gender identity fueled parental protests outside primary schools that had adopted an LGBTQ+ curriculum. Subsequent government guidance in England to support schools with the advancement of LGBTQ+ equality has been weak and arguably this has demonstrated a lack of political commitment to equality.

This paper uses Meyer's model of minority stress (Meyer's, 2003) as a conceptual lens to support the analysis of the policy. As a conceptual lens, this model is particularly useful in that it helps to frame the experiences of individuals with minority identities. For example, LGBTQ+ youth may be exposed to a range of stressors both in society and in school and these can impact on their ability to thrive within educational environments and lead to mental ill health (Meyer's, 2003). The model identifies that individuals with minority identities are exposed to two additional stressors in addition to the general stressors that everyone experiences; distal stressors are the direct experience of prejudice and discrimination as a direct result of one's minority identity. Proximal stressors occur when individuals anticipate that they will be exposed to distal stressors which can result in concealment of one's identity and internalized homophobia (Meyer's, 2003). This paper argues that an inclusive relationships and sex education curriculum, which provides validation and positive affirmation of different identities, has the potential to reduce minority stress in young people who have non-normative gender identities and sexual orientations. In addition, we argue that government policy of delaying the introduction of inclusive relationships education will potentially increase minority stress in young people with these minority identities. We therefore argue that a curriculum which addresses inclusive relationships and sex education is a useful tool for reducing the effects of minority stress in LGBTQ+ youth.

## Policy Context in England

Sexual orientation and gender identity are two crucial components on an individual's identity, although the Equality Act (2010) in England specifically refers to “gender reassignment.” In England, sexual orientation and gender reassignment are identified as “protected characteristics” in the Equality Act (2010). Schools and other public institutions must therefore ensure that LGBTQ+ individuals are protected from both direct and indirect forms of discrimination. In addition, the Public Sector Equality Duty (Section 149 of the Equality Act, 2010) requires schools to advance equality of opportunity between individuals with and without protected characteristics and to foster good relations between these two groups.

In 2017 the UK Prime Minister, Theresa May, delivered a keynote speech at the Pink Awards:

*Homophobia, biphobia and transphobia have still not been defeated and they must be. Bullying in schools and on social media is still a daily reality for young LGBTQ*+ *people, and that has to stop. Trans people still face indignities and prejudice when they deserve understanding and respect… being trans is not an illness and it shouldn't be treated as such*.

She emphasized the importance of introducing inclusive relationships and sex education into Britain's schools. Of course, 2017 also marked 50 years following the partial decriminalization of homosexuality through the 1967 Sexual Offences Act. The direction of travel was a stark contrast to Section 28 in 1988 which was introduced by the former Conservative Prime Minister, Margaret Thatcher. Section 28 was a controversial piece of legislation which stated that local authorities “shall not intentionally promote homosexuality or publish material with the intention of promoting homosexuality or promote the teaching in any maintained school of the acceptability of homosexuality as a pretended family relationship.” It silenced schools from discussing homosexuality and forced LGBTQ+ teachers further into the closet. Section 28 was eventually repealed in 2003. However, its existence demonstrated the role of educational policy in maintaining a dominant heteronormative discourse, thus leading to the marginalization of LGBTQ+ people.

The parental protests in England in 2019 also demonstrated how religious beliefs can also seek to maintain discourses of heteronormativity and also highlighted the ways in which one protected characteristic (religion or belief) can clash with other protected characteristics (gender reassignment and sexual orientation). During these protests, parents objected to LGBTQ+ curricula in primary schools on the grounds that this curriculum was in direct conflict with religious beliefs. Following a protest at a school in Birmingham, these were repeated in other schools in other parts of the UK. These examples of resistance serve to demonstrate the controversial nature of this topic and in particular the apparent tensions between religion, sexuality and gender identity. However, despite these objections it is important that schools leaders respect different opinions, and religious beliefs, but also explain to parents why it is necessary for all young people to learn about different types of relationships and family structures.

It should be emphasized that the statutory guidance for relationships and sex education (Department for Education, 2019) does not seek to promote a particular lifestyle. An effective LGBTQ+ curriculum enables children and young people to know that LGBTQ+ people exist and that it is legal to be LGBTQ+. It supports them to understand different family structures and to know that under the rule of British law it is legal to both enter into same-sex relationships and get married. It is critically important that all children are taught to respect all forms of difference. It is also important to acknowledge to young people that although LGBTQ+ identities and relationships may not be permitted within the context of a religion, in the UK they are permitted under the rule of law. Given that LGBTQ+ people exist within all walks of life (in families, schools, colleges, universities, the workplace, and the community) it is important that young people learn to respect people's differences, regardless of personal or religious beliefs. Education should play a critical role in supporting all children and young people to understand that prejudice and discrimination are wrong, both from a legal and a moral perspective. Critical pedagogy serves a powerful role in advancing social justice through educating young people about all forms of discrimination. It offers hope for creating a better and more equitable society in the future and supports young people to be responsible future citizens.

Research has found that LGBTQ+ policies and initiatives in schools which promote queer-straight alliances are distinctly and mutually important for fostering safer and more supportive school climates for young people and may reduce prejudice-based bullying (Poteat et al., 2013; Ioverno et al., 2016; Day et al., 2019). Lessons which address inclusive relationships and sex education are one example of these alliances. Creating safe spaces in which all young people can discuss inclusive relationships may therefore play a critical role in fostering positive attitudes, creating positive school cultures and reducing homophobic, biphobic, and transphobic bullying. Research by Russell et al. (2009) found that safe queer-straight alliances led to three inter-related dimensions of empowerment: personal empowerment, relational empowerment, and strategic empowerment. When these three dimensions are experienced in combination, teachers of inclusive relationships and sex education can facilitate individual and collective empowerment which can lead to social change in schools.

## A Critical Analysis of the Statutory Guidance

In 2019 the Department for Education (DfE) published statutory guidance for the teaching of inclusive relationships and sex education in schools in England. The DfE is a government organization that enforces policy in schools. The guidance replaced previous guidance which was published 20 years earlier and schools in England are required by law to implement the guidance from 2020. Schools which do not implement the statutory guidance will face penalties during school inspections. The guidance was refreshed to address current societal issues and addresses topics such as consent, domestic abuse and online relationships. It also explicitly mandates the teaching of LGBTQ+ identities and relationships in primary and secondary schools. However, in relation to LGBTQ+ content the guidance might be interpreted in ways which effectively permit schools to opt out of delivering this content, particularly to younger children. The quotations from the guidance below particularly provide schools with a rationale for not delivering LGBTQ+ related content, despite the statutory nature of the guidance. We argue that these opt-out clauses are not acceptable and may potentially result in LGBTQ+ identities not being validated or positively affirmed.

The Relationships and Sex Education States:

- In all schools, when teaching these subjects, **the religious background of all pupils must be taken into account** when planning teaching, so that the topics that are included in the core content in this guidance are appropriately handled. Schools must ensure they comply with the relevant provisions of the Equality Act (2010), under which religion or belief are amongst the protected characteristics (Department for Education, 2019, para, 20, p. 12).
- In particular, **schools with a religious character may teach the distinctive faith perspective on relationships**, and balanced debate may take place about issues that are seen as contentious (Department for Education, 2019, para, 21, p. 12).
- Schools should ensure that all of their **teaching is sensitive and age appropriate** in approach and content (Department for Education, 2019, para, 37, p. 15).

In response to the parental protests, the Department for Education (DfE) introduced the following guidance for schools:

- In all schools, when teaching Relationships Education, **the age and religious background of all pupils must be taken into account** when planning teaching (Department for Education, 2020a, p. 11).

We have added emphasis to the text to draw attention to some key concerns. Schools will not be compliant with the Equality Act (2010) if young people are not taught to respect different religious beliefs. However, there is a danger that schools with a religious character will use these statements to avoid including LGBTQ+ identities and relationships into the curriculum. It is worrying that the policy permits schools with a religious character to teach “distinctive faith perspectives on relationships” given that some of these perspectives may not align with the principles of the Equality Act (2010). It is also a concern that the teaching of LGBTQ+ relationships and identities is acknowledged within the policy framework as a “sensitive” aspect of the curriculum. This phrasing is unhelpful because it further stigmatizes LGBTQ+ individuals whose identities should be validated and celebrated. The phrase “age-appropriate” is also potentially damaging. It suggests that younger children need to be somehow protected from this content, thus suggesting that it may be potentially harmful and damaging. LGBTQ+ people exist within families and communities. Young children in nursery schools may have same-sex parents, siblings or members of their wider family who are LGBTQ+. To deliberately avoid addressing this in the early years is likely to lead to young children in same-sex families or those with LGBTQ+ family members feeling excluded. This does not foster a sense of belonging and it does not provide validation of children's families particularly in cases where children have LGBTQ+ parents or siblings.

From 1 September 2020, relationships education is compulsory for all primary school pupils and relationships and sex education (RSE) is compulsory for all secondary school pupils (Department for Education, 2020b). However, as a result of the impact of Covid-19 schools have been given additional time to implement the statutory guidance. The government has insisted that secondary schools will risk negative inspection reports if the statutory guidance is not implemented from the start of the summer term 2021. In stark contrast, primary schools will not be penalized for avoiding the teaching of LGBTQ+ content, provided that they can demonstrate that appropriate consultation has taken place with parents:

*Before the start of summer term 2021, if a primary school does not teach about LGBT relationships, and does not yet have adequate plans in place to meet the requirements of the DfE's statutory guidance by the start of the summer term 2021 (for example, if it has not consulted parents and has no plans to do so before then), inspectors will comment on this in the inspection report. This will not, however, impact on the leadership and management judgement except when inspectors consider it relevant to the effectiveness of the school's safeguarding arrangements (Department for Education, 2020b)*.

*From the start of summer term 2021, if a primary school does not teach about LGBT relationships, this will not have an impact on the leadership and management judgement as long as the school can satisfy inspectors that it has still fulfilled the requirements of the DfE's statutory guidance. If it cannot do this, for example if it has failed to consult with parents, inspectors will consider this when making the leadership and management judgement. The school will not ordinarily receive a judgement for this better than requires improvement (Department for Education, 2020b)*.

*Before the start of summer term 2021, if a secondary school does not teach about LGBT relationships and does not have adequate plans in place to meet the requirements of the DfE's statutory guidance by the start of the summer term 2021, inspectors will comment on this in the inspection report. This will not, however, impact on the leadership and management judgement except when inspectors consider it relevant to the effectiveness of the school's safeguarding arrangements (Department for Education, 2020b)*.

*From the start of summer term 2021, if a secondary school does not teach about LGBT relationships, it will not be meeting the requirements of the DfE's statutory guidance. Inspectors will consider this when making the leadership and management judgement. For state-funded schools, this only applies to section 5 inspections. For independent schools, this only applies to standard inspections. The school will not ordinarily receive a judgement for this better than requires improvement (Department for Education, 2020b)*.

Given that prejudice is often established before children start the secondary phase of their education, we feel that it is critical that the teaching of LGBTQ+ content in primary schools should be mandatory. This latest “opt out clause” permits parental beliefs (and parental prejudice) to determine curriculum content. This is not only selling LGBTQ+ pupils in primary schools short, it is also selling all pupils short. It effectively provides schools that are reluctant to address this content with a license not to address it. Large-scale survey data from Stonewall in 2017, the organization which champions equality for the LGBTQ+ community, demonstrates the extent of homophobic bullying in Britain's schools. The data demonstrated the large prevalence of homophobic, biphobic, and transphobic bullying in Britain's schools. We argue that inclusive relationships and sex education in primary and secondary schools which provides positive affirmation of different identities will reduce the prevalence of prejudice-based bullying.

## Theoretical Critique

Meyer's (2003) minority stress model has been used by mainstream psychologists to explain how minority status can impact on mental health outcomes for individuals who identify as part of a minority group. The model has been applied to individuals who identify as LGBTQ+.

The model identifies different types of stress that minority individuals experience. These are summarized below:

- General stressors apply to all individuals as a result of environmental circumstances.
- Distal stressors: the direct experience of stigma, prejudice, discrimination, victimization and bullying by others based on an individual's minority status produces distal stressors. These experiences can be shaped by structural forces (for example, racism, heteronormativity/heterosexism) which result in structural disadvantage for minority groups.
- Proximal stressors: these relate to an individual's perception or appraisal of situations. The expectation or anticipation that a person with a minority status may experience rejection, discrimination, victimization, or stigmatization based on one's previous experiences of this can result in self-vigilance and identity concealment. People who identify as LGBTQ+ may anticipate negative reactions to their sexual orientation or gender identity in specific situations due to their previous negative experiences. To reduce the likelihood of negative experiences occurring, self-vigilance and concealment are employed but these tactics can result in fear of discovery, psychological distress, internalized shame, guilt, anxiety, and social isolation.

Not addressing LGBTQ+-related content in the primary curriculum is likely to result in exposing children to distal and proximal stressors. If their identities are not discussed and not made visible through the school environment and the curriculum, they are more likely to conceal their identities and to internalize the homophobia to which they are exposed. The aim of an LGBTQ+ curriculum is to validate identities of difference and to teach children the importance of respect. If this validation of identities is not evident, there is a risk that children with non-normative identities in primary schools will be exposed to prejudice, violence and other forms of discrimination.

International research continues to demonstrate that heteronormative and heterosexist cultures are entrenched within schools (Kjaran and Kristinsdóttir, 2015). Even in countries known for their liberal attitude toward sexuality, such as Sweden, heteronormative attitudes continue to prevail within schools (Lundin, 2015). The revision of policies and legislation signal the UK government's commitment to LGBTQ+ inclusion (DePalma and Jennett, 2010). However, despite this, research continues to evidence the scale of homophobic, biphobic, and transphobic bullying in Britain's schools (Bradlow et al., 2017). Whilst the reasons for this are complex, multifaceted, and often misunderstood (Formby, 2015), research by Bradlow et al. (2017) does illuminate the disconnect between the government's expectations and the lived experiences of those within the LGBTQ+ community.

## Conclusion

Data from Stonewall (Bradlow et al., 2017) demonstrates the prevalence of homophobic, biphobic and transphobic bullying in schools in Britain. Nearly half of lesbian, gay, bi and trans pupils (45%)—including 64% of trans pupils—are bullied for being LGBTQ+ at school. The majority of LGBTQ+ pupils-−86%—regularly hear phrases including “that's so gay” or “you're so gay” in school. Nearly one in 10 trans pupils (9%) are subjected to death threats at school. Seven in 10 LGBTQ+ pupils (68%) report that teachers or school staff only “sometimes” or “never” challenge homophobic, biphobic, and transphobic language when they hear it. Two in five LGBTQ+ pupils (40%) are never taught anything about LGBTQ+ identities at school. Three in four LGBTQ+ pupils (77%) have never learnt about gender identity and what “trans” means at school. More than half of LGBTQ+ pupils (53%) say that there isn't an adult at school they can talk to about being LGBTQ+. Two in five pupils who have been bullied for being LGBTQ+ (40%) have missed school because of this bullying. Half of bullied LGBTQ+ pupils (52%) feel that homophobic, biphobic, and transphobic bullying has had a negative effect on their plans for future education. More than four in five trans young people (84%) have self-harmed. For lesbian, gay, and bi young people who aren't trans, three in five (61%) have self-harmed. More than two in five trans young people (45%) have attempted to take their own life. For lesbian, gay, and bi young people who aren't trans, over one in five (22%) have attempted to take their own life (Bradlow et al., 2017).

Avoiding teaching LGBTQ-related content in primary schools is likely to result in a worsening of these statistics. In addition, many young children in primary schools have same-sex parents or they may have siblings or know other people who are LGBTQ+. Silencing LGBTQ+ identities is likely to alienate these children if they start to feel that their daily realities are not reflected in the school environment or through the curriculum that they are taught. Avoiding teaching LGBTQ+ related content to young children is likely to result in minority stress and mental ill health (Meyer's, 2003), especially if queer identities are not recognized, not provided with validation and not positively affirmed.

## Actionable Recommendations

The following recommendations are aimed at school leaders:

- All primary and secondary schools should provide children with an inclusive relationships education curriculum which addresses LGBTQ+ identities and same-sex relationships.
- All primary and secondary schools should teach children to respect LGBTQ+ people.
- All schools should consult with parents in relation to LGBTQ+-related content but consultation should not lead to a veto on the curriculum.
- Penalties should be applied by the school inspectorate to primary schools that do not teach children about LGBTQ+-related content.
- All schools should ensure that their legal obligations in relation to the Equality Act (2010) are met.
- All schools should have a clear policy which addresses LGBTQ+ inclusion.

## Author Contributions

JG outlined the policy context and offered a theoretical critique. SS contributed the review of statutory guidance. All authors identified recommendations and edited and approved the article.

## Conflict of Interest

The authors declare that the research was conducted in the absence of any commercial or financial relationships that could be construed as a potential conflict of interest.
